# Engaging the Canadian public on reimbursement decision-making for drugs for rare diseases: a national online survey

**DOI:** 10.1186/s12913-017-2310-4

**Published:** 2017-05-26

**Authors:** Julie Polisena, Michael Burgess, Craig Mitton, Larry D. Lynd

**Affiliations:** 1 Canadian Agency for Drugs and Technologies in Health, 600-865 Carling Ave, Ottawa, K1S 5S8 ON Canada; 2 W. Maurice Young Centre for Applied Ethics, School of Population and Public Health, University of British Columbia, Kelowna, BC Canada; 3 School of Population and Public Health, University of British Columbia, Vancouver, BC Canada; 4 Centre for Clinical Epidemiology and Evaluation, Vancouver, BC Canada; 5 Faculty of Pharmaceutical Sciences, University of British Columbia, Vancouver, BC Canada; 6 Centre for Health Evaluation and Outcomes Sciences, Providence Health Research Institute, Vancouver, BC Canada

**Keywords:** Drugs, Reimbursement, Rare diseases, Survey, Public engagement

## Abstract

**Background:**

Funding of drugs for rare diseases (DRDs) requires decisions that balance fairness for all individuals within the healthcare system with compassion for affected individuals. Our study objective was to conduct a national online survey to determine the Canadian public’s perspective, including regional variations, associated with DRD decision-making.

**Methods:**

The survey collected responses from 1631 Canadians. Respondents were asked to rank at least three and up to five DRD decision-making priorities, out of a total of eight priorities presented. They were also asked to compare and rate their agreement level on a 5-point Likert scale with four funding scenarios described. The frequency of each priority, independent of where it was ranked in relation to the other priorities, was calculated. Regression analyses were conducted to measure the association between respondents’ demographics and selected priorities with their agreement level for each funding scenario.

**Results:**

Among the survey respondents, *Improved Quality of Life* and *Effective Health Care* were most frequently selected as top priorities. Also, 79.2% of respondents agreed with equal access to DRDs across Canada, and 73.0% agreed with DRD funding if additional expenses are justified in the DRD’s cost-effectiveness. Approximately half agreed to pay for DRDs independent of their effectiveness. There were no geographic differences in priorities.

Selecting *Effective Health Care* in the top priorities was positively associated with both prioritizing other programs over programs for rare diseases and DRD funding only if deemed as cost-effective. Respondents, who selected *National Access* as one of the top priorities, were less likely to agree to fund DRDs only if deemed as cost-effective and were more likely to agree with the scenario to provide national access to DRDs.

**Conclusions:**

The survey results suggest the level of public support for funding decisions and programs that incorporate assessment of the effectiveness of drugs for improving quality of life, and to promote similar access across Canada. The responses anticipate public responses to different policy scenarios and the priorities that underlie them. Decision-makers may find it useful to consider whether and how to incorporate these results into policy decisions and their justification to citizens and patients.

**Electronic supplementary material:**

The online version of this article (doi:10.1186/s12913-017-2310-4) contains supplementary material, which is available to authorized users.

## Background

Funding of expensive drugs for rare diseases (DRDs) requires decisions that balance fairness for all individuals within the healthcare system with compassion for individuals with rare diseases. As there is no universal definition of a rare disease, we applied the average prevalence used in Canada (38 cases per 100,000 people; minimum = 1 and maximum = 50 across jurisdictions) [1]. In Canada’s publicly funded healthcare system, the multiple processes to assess reimbursement of DRDs are generally comparable, but access to specific drugs differs across jurisdictions. The variation can, in part, be attributed to the prevalence of the rare disease in specific provinces or territories [2]. For instance, Alberta defines a rare disease as a genetic lysosomal storage disorder that is prevalent in fewer than 1 per 50,000 Canadians, while Ontario and Saskatchewan define it as a disease with an incidence rate of fewer than 1 in 150,000 live births or new diagnoses annually, and British Columbia consider a rare disease with a prevalence as fewer than 1.7 per 100,000 [2, 3]. The availability of high quality evidence is a challenge due to the low prevalence of these diseases, and manufacturers typically assign high prices to recover research and production related costs [4].

Even though an increasing number of countries have implemented regulations for the development and market approval for DRDs, Canada does not have national policies or a pan-Canadian legislative framework in place or a reimbursement review process for DRDs. They do, however, exist in five provinces: British Columbia, Alberta, Saskatchewan, Ontario, and New Brunswick [3]. Unlike other provinces with DRD programs, a coverage decision will impact access to the drug for all patients with the rare disease in Ontario, and not an individual patient. Across the programs, a panel or committee that includes at least one disease expert or specialist will review an application to understand the disease severity and alternative treatments, assess the potential effectiveness, or evaluate the budget and cost impact. Submissions by patient groups can also be reviewed. Recommendations will usually include options for coverage with conditions or do not provide coverage. Coverage with conditions usually implies continued monitoring for clinical outcomes or the patient’s response to treatment [3]. Similar to Canada, most 20 OECD countries with a socially funded health care system or universal health care do not have a national program for funding DRDs, except for the United Kingdom. Instead, many apply a “safety net” program or use modified decision criteria when their common drug review processes are insufficient for funding decisions for DRDs [5].

In 2012, Health Canada presented a draft framework for orphan drug regulatory approval, engaged in a pan-Canadian discussion, and posted it online for comments. The framework aims to develop and implement a comprehensive approach meant to support industry in their submission for market access approval without compromising patient safety. It is based on an integrated approach that incorporates information collected from patients, health care professionals, researchers, payers, and international regulators to enhance the quality of knowledge for decision making and reduce uncertainty. Similar to DRD legislation established by other international regulatory agencies, key components of the framework involve orphan drug designation, regulatory and expert advice and patient representation from Health Canada, and increased transparency in the market authorization process [6]. At the time of our study, the framework had not been finalized.

Paulden et al. conducted a scoping review published in 2015 to identify the medical and grey literature to understand the societal values associated with DRD reimbursement, what potential role they can play, and outlined a decision-making framework that identifies how societal values can be introduced to guide discussions and increase transparency on reimbursement decisions. The authors recognized that there are many places in the decision-making framework where public values are relevant [4].

Dionne et al. developed a framework to operationalize a multi-criteria decision analysis (MCDA) method that incorporates societal values on drug reimbursement decisions as an alternative to cost-effective analysis [7]. MCDA is used to primarily measure value based on numerous criteria. According to the results of two workshops held in Canada with pharmaceutical, industry, patient, healthcare, and decision-making representatives, ten criteria were identified. They were as follows: comparative effectiveness, adoption feasibility, risks of adverse events, patient autonomy, societal benefit, equity, strength of evidence, incidence/prevalence/severity of condition, innovation and disease prevention/health promotion [7]. In addition to drug reimbursement decisions for common diseases, the authors suggest that this framework is applicable to funding DRDs. At the time of publication, the proposed framework had not been finalized.

Public engagement helps to inform decision-makers about the diverse perspectives of the Canadian population, including differences among stakeholder, patient, and citizen perspectives [8]. Since it is necessary to consider the fair distribution of health services or funds across health needs and how that is to be weighed against access to DRDs, it is important to include citizens or public whose interests are outside the access to DRDs. This broad representation needs to be considered when the trade-offs between providing access for DRDs are weighed against other possible uses (i.e., the opportunity cost) of the health care funds. Wider representation may add legitimacy to decisions and policies by involving diverse citizenry on what counts as fair and reasonable and by adding transparency to decision-making processes [9–11].

Survey approaches to identify the perspectives of respondents tend to characterize their views based on a small amount of information. Surveys can be distinguished from deliberative approaches that emphasize well-informed and civic-minded input of diverse, small groups, such as citizen juries and study circles. Careful informing and facilitation of small group deliberation produces advice that balances the diversity of interests to deliver civic-minded recommendations directed to specific decisions or trade-offs. Nevertheless, public views aggregated from a survey reflect help decision makers understand how a wider public might respond to policy changes. They many also be used to inform future deliberative engagement.

The main objective of this study was to engage a diverse sample of Canadians to determine their perspectives, including any regional variations, associated with DRD decision-making, as determined through a national online survey. The responses provided insight into public perspectives related to DRD funding that decision-makers might want to consider when reviewing policies and determining the allocation of scarce resources. This study was reviewed and approved by the University of British Columbia’s Behaviour Research Ethics Board (UBC BREB number H15-02972).

## Methods

### Survey questionnaire

The survey sought to characterize a sample of Canadians’ priorities and perspectives associated with decision-making for DRDs. An online survey, available in English and French, was administered in January and February 2016 (Additional file 1).

The priorities and scenarios in the survey were developed over 2 years of collaboration with researchers and decision makers involved in provincial drug funding decisions and Canadian federal recommendations, and with the Canadian Institutes for Health Research (CIHR) New Emerging Team for Rare Diseases [12]. Supporting respondents’ ability to identify their priorities and receive descriptions of how the priorities would be promoted or mitigated by the scenarios provided some decision-support that is a rough parallel with the structure of a deliberation. This approach moved from the identification of participants’ hopes and concerns to assessing how these were affected by different policy choices. The survey design by MetroQuest encouraged respondents to think about priorities in relation to DRD funding decisions, and provided estimates of how the each respondent’s selected priorities would be affected by each of the funding scenarios. This structure aimed to guide respondents through a basic understanding of the policy challenge, identify their priorities, and relate those priorities to policy decisions in a survey intended to take less than 10 min. The final version of the survey was reviewed and revised by the CIHR New Emerging Team [12].

Survey respondents were asked to rank priorities, and then use the priorities to indicate their degree of support for the funding scenarios. This method may not achieve the degree of reflection and reason-giving typical of a deliberative engagement, but it provides an opportunity for analysis to assess the priorities in relation to the scenarios [9, 13].

Respondents ranked, in order of importance to them, at least three and up to five DRD decision-making priorities, out of a total of eight potential priorities listed in the survey. The eight priorities presented included *Pain Reduction*, *Improve Quality of Life*, *Longer Life*, *National Equal Access*, *Lack of Current Treatment*, *Severity of Symptoms*, *Cost Containment*, and *Effective Health Care*. They were also asked to compare and rate their agreement level with each scenario using a 5-point Likert scale, ranging from 1 = completely disagree to 5 = completely agree. Each scenario represented a different proposition associated with funding for DRDs. The titles of the scenarios were: *Fund Drug if Justified*, *Canadawide Equality*, *Pay for Drugs*, and *Prioritize Other Programs*. Table 1 describes the full description of each scenario as included in the survey.

**Table 1:** Description of each funding scenario

Funding Scenario	Description in Survey
Pay for Drugs	Provincial decision-makers fund new expensive drugs. They do not consider alternative uses of funds but argue that ANY improvement for patient care must be funded, even if that means that new programs cannot be started. This gives high priority to all benefits to patients with rare diseases, at the expense of funding to other programs and patients. It does not address health care budgets or provincial differences.
Prioritize Other Programs	Provincial decision-makers do not fund access to new expensive drugs. Instead, funding is allocated to programs that target reduced sickness and death in underserved populations, often through access to basic health care. Treatment for patients with rare diseases are only funded when they are highly effective and not too expensive. Health care budgets are contained. Provincial differences are not equalized.
Fund Drug if Justified	Fund the drug ONLY if the extra expense is well justified. Effective health care and cost containment are promoted. Priorities related to patients with rare diseases are promoted only when they compare well to other uses of funds and significantly improve treatments that are currently available. Provincial differences in funding are not changed.
Canadawide Equality	All provincial and territorial Ministries of Health are required by a new law to use recommendations of a federal drug assessment agency. Canadians receive the same access across the provinces and territories. Benefits for patients with rare diseases are promoted when they compare well to alternative use of the funds and are significant improvements over current treatments. This supports assessing cost containment and effective health care on a national level.

Respondents were able to view an estimate of how each funding scenario agreed or disagreed with their selected priorities. Providing estimates of how each scenario promoted or compromised the priority selection was a decision support that helped survey respondents to more efficiently consider the scenarios based on their selected priorities. It did not, however, preclude them from rating the funding scenarios using a 5-point Likert scale independently of the projected effect on their selected priorities. Prior to submitting the survey, respondents had the opportunity to revise any of their responses in previous screens and to submit comments in an open-ended question with regards to DRD funding.

### Survey sample

A third-party organization, Asking Canadians, was responsible for recruiting the survey sample of approximately 1600 individuals from across Canada. Respondents were solicited from AskingCanadians’ panel. The company had several quality assurance checkpoints to ensure the integrity of the survey respondents. For our study, once AskingCanadians’ panel members were recruited, they were asked to confirm their desire to participate in the survey before being redirected to the MetroQuest survey. A survey was classified as complete if at least three priorities were ranked, and all four scenarios were rated. Demographic data was also collected for each respondent which included age, annual household income, geographic region, sex, and education level.

### Survey data analysis

#### Descriptive statistics

Survey respondents were asked to rank at least three and up to five DRD decision-making priorities in order of importance from their own personal perspective. We determined the proportion of respondents that ranked each of the priorities (i.e., selected in their top three to five priorities), independent of where it was ranked in relation to the other priorities.

Respondents’ agreement with each funding scenario was summarized into two categories: agree (i.e., 4 = agree and 5 = completely agree) and do not agree (i.e., 1 = completely disagree, 2 = disagree, and 3 = neither agree nor disagree). Descriptive statistics were used to present the frequency of the individual priority selected, agreement level with each scenario and respondents’ demographics.

#### Logistic regression analyses

Univariate analyses were performed to measure the association between respondent demographics and the selected priorities with respondents’ agreement with each funding scenario using logistic regression analyses. For each regression analysis, the dependent variable was the agreement level with a specific funding scenario, and the reference category was “Do not agree”. Demographics and priority selection with *p*-values less than 0.05 from the univariate analyses were included in multiple logistic regression analyses to obtain the association between funding scenarios and demographics and priority selection. All analyses were conducted using RStudio® (Version 0.99.878, Boston, USA).

#### Content analysis on open-ended question on funding for expensive drugs

Content analysis, a qualitative research method, was conducted to review and categorize the open-ended responses submitted by 410 survey respondents [14, 15]. The responses were reviewed, defined and organized into themes. An emergent list of codes of distinct ideas was maintained, as well as their definitions and a sample text passage that illustrates the application of each code. The codes were reviewed using a constant comparative technique to identify all instances and appropriateness of the coding framework, and to determine how to expand or merge them into themes. The responses were reviewed again to ensure that all the relevant information was coded. The themes derived from the content analysis were summarized narratively.

## Results

### Descriptive statistics

Table 2 presents the demographics of the 1631 respondents who completed the survey. An even proportion of males and females participated in the survey, and the mean age was 48.0 years old (standard deviation = 16.0 years). Close to 60% of respondents had completed college or university, obtained a post-graduate degree, or had a professional degree. Over 25% had an annual household income of more than CDN$100,000, and 22.9% of respondents (*n* = 373) preferred not to reveal their income. Among the 1631 respondents, Alberta (*n* = 162; 9.9%), Manitoba (*n* = 157; 9.6%), and Saskatchewan (*n* = 145; 8. 9%) represented the greatest proportion of respondents across all provinces, and 32.7% of respondents (*n* = 533) preferred not identify their location.

**Table 2:** Survey respondent characteristics

Characteristic	Frequency (*N* = 1631) (%)	Mean (SD)
*Age (in years)*		48.0 (16.0)
*Age Category (in years)*		
18–34	448 (27.5)	
35–50	536 (32.9)	
51–75	545 (33.4)	
76–100	97 (6.0)	
No response	5 (0.3)	
*Sex*		
Female	819 (50.2)	
*Education Level*		
Less than Grade 9	10 (0.6)	
Grade 9 to Grade 13	179 (11.0)	
Trades certificate or diploma	139 (8.5)	
Some college or university	268 (16.4)	
Completed college or university	662 (40.6)	
Post-graduate or professional degree	310 (19.0)	
Prefer not to say	61 (3.7)	
No response	2 (0.1)	
*Annual Household Income*		
Less than $20,000–$44,999	254 (15.6)	
$45,000–$99,999	563 (34.5)	
$100,000–$499,999	416 (25.5)	
$500,000–$1,000,000+	9 (0.6)	
Prefer not to say	373 (22.9)	
No response	16 (1.0)	
*Province of Residence*		
British Columbia	138 (8.5)	
Alberta	162 (9.9)	
Saskatchewan	145 (8.9)	
Manitoba	157 (9.6)	
Ontario	130 (8.0)	
National Capital Region^a^	20 (1.2)	
Quebec	130 (8.00)	
Nova Scotia	55 (3.4)	
New Brunswick	42 (2.6)	
Prince Edward Island	6 (0.4)	
Newfoundland and Labrador	113 (6.9)	
Preferred not to say	533 (32.7)	

^a^Includes Ottawa and Gatineau; *SD* standard deviation

Surveys that ranked at least three priorities and rated all scenarios were labelled as complete, but 24 completed surveys were missing the scenario ratings. This figure represents 1.47% of responses, so the impact on the analyses was minimal.

The priorities that were most frequently selected by respondents as being important when considering funding of drugs were *Improve Quality of Life* (*n* = 1304; 80.0%) and *Effective Health Care* (*n* = 1232; 75.5%). The third most frequently selected priority was *National Equal Access* (*n* = 701; 45%). Less than 25% of respondents selected *Lack of Current Treatment* (*n* = 372; 22.8%) and *Longer Life* (*n* = 376; 23.1%) (Table 3).

**Table 3:** Frequency of priority selection and agreement level for funding scenarios (*N* = 1631)

Priority Selection		
Description	Frequency (%)	
Improve Quality of Life	1304 (80.0)	
Effective Health Care	1232 (75.5)	
National Equal Access	701 (43.0)	
Pain Reduction	642 (39.4)	
Cost Containment	543 (33.3)	
Severity of Symptoms	430 (26.4)	
Longer Life	376 (23.1)	
Lack of Current Treatment	372 (22.8)	
Funding Scenario by Agreement Level		
Description	Agreement Level	Frequency (%)
Canadawide Equality	Agree	1292 (79.2)
	Neither agree nor disagree	229 (14.0)
	Disagree	95 (5.8)
	No response	15 (0.9)
Fund Drug if Justified	Agree	1190 (73.0)
	Neither agree nor disagree	311 (19.1)
	Disagree	115 (7.1)
	No response	15 (0.9)
Pay for Drugs	Agree	818 (50.2)
	Neither agree nor disagree	484 (29.7)
	Disagree	305 (18.7)
	No response	24 (1.4)
Prioritize Other Programs	Agree	601 (36.8)
	Neither agree nor disagree	549 (33.7
	Disagree	460 (28.2)
	No response	21 (1.3)

Agree: 4 = agree and 5 = completely agree; Disagree: 1 = completely disagree and 2 = disagree; Neither agree nor disagree: 3 = neutral

Among the four scenarios presented, over 75% of respondents agreed with the scenario that described a funding model that grants equal access to treatment to all Canadians, independent of where they live, followed by 73.0% who agreed that a DRD should be funded if deemed to be cost-effective. Moreover, half of respondents support DRD funding to improve patient care for rare diseases, while over 35% agreed that provincial funding should be allocated to programs that target reduced sickness and death in underserved populations instead of funding DRDs.

### Logistic regression analyses

In our initial analyses, we evaluated the relationship between agreement with each funding scenario and respondent demographics including geographical location. See Additional file 2 for frequency of priority ranking and ratings of scenarios by province. We did not, however, find any significant associations between demographic characteristics and respondents’ agreement with any specific scenario.

Table 4 presents the results of multiple logistic regression analyses that included variables in which the association between the funding scenarios and individual priority selections were statistically significant. As there was no association between any demographic characteristics and the level of agreement with each scenario, only the relationship between priority and agreement level with the scenarios were modelled.

**Table 4:** Association between priority selection and agreement with each funding scenario

Funding Scenario^a^	Priority	OR	95% CI
Pay for Drugs^b^	Longer Life	1.52	1.20–1.93
	Cost Containment^b^	0.71	0.58–0.88
Prioritize Other Programs	Effective Healthcare	1.42	1.17–1.82
	Cost Containment	1.67	1.34–2.06
	Lack of Treatment	0.78	0.61–1.00
Fund Drug if Justified	Effective Healthcare	1.55	1.21–1.98
Canada-wide Equality	Longer Life	1.57	1.14–2.20
	Cost Containment	0.50	0.38–0.65
	National Access	4.14	3.07–5.65
	Severity of Symptoms	0.70	0.53–0.93

^a^Reference category = Do not agree

^b^Interpretation example: Respondents who ranked cost containment as one their top priorities were 29% less likely (OR 0.71) to agree that provincial payers should pay for DRDs if there is any improvement in health, without considering the potential uses of these funds. Odds ratios represent the odds of agreeing or strongly agreeing with the scenario if that priority was selected as a top priority versus if it was not

Agree: 4 = agree and 5 = completely agree; CI: confidence interval; Do not agree: 1 = completely disagree, 2 = disagree, and 3 = neither agree nor disagree; OR = odds ratio

The selection of *Cost Containment* as one of the top priorities was associated with the level of agreement on three of the four funding scenarios. Specifically, those who selected *Cost Containment* in their top priorities were less likely to agree with paying for all drugs for rare diseases and Canada-wide equality of drug access versus those who did not select this priority. Respondents, who ranked *Cost Containment* in their top priorities, were also 67% more likely to agree with the prioritization of other programs over programs for rare diseases. Those who selected *Longer Life* were more likely to agree with both paying for drugs for rare diseases as a matter of principle, and the necessity of Canada-wide equality for access to treatment. Selecting *Effective Health Care* in the top priorities was positively associated with both prioritizing other programs over programs for rare diseases, and only funding a drug treatment if justified. As well, respondents, who selected *National Access* as one of the top priorities, were four times more likely to agree with the scenario to provide equal access to drugs across Canada.

### Content analysis on open-ended question on funding for expensive drugs

Respondents had the opportunity to submit their comments on DRD funding. Among the 1631 respondents who completed the survey, 410 responded to the open-ended question (25.14%).

Several themes that emerged from the comments submitted on funding for expensive drugs were aligned with the priorities listed in the survey, such as national access to DRDs and cost containment. Other themes captured in the comments were directly related to the patient’s well-being and welfare. They included the financial burden imposed on patients and consideration for their age and autonomy. For example, several respondents wrote that families experience a heavy financial burden when drugs are not funded, and individuals should not have to choose between paying for drugs for their health versus paying for their food. Respondents also commented on drug coverage decisions based on cost-effectiveness versus decisions driven by compassion for the patient, and they questioned how the value of life was measured. The impact of media coverage on the individual cases and ensuring the accountability of industry, who charge high costs for DRDs, were highlighted as concerns by numerous respondents. For instance, one respondent wrote that decisions should be based on real data and not on emotions or “over-the-top” media-coverage. Themes identified also focused on the balance between funding for DRDs compared with funding drugs for common diseases that affect a larger population, coverage through private insurance versus public sources, and increased frequency of bulk purchases to obtain large volume discounts. One respondent commented that a national drug program was needed, and another respondent suggested a Canada-wide purchase scheme to reduce the cost of drugs. Additional themes that surfaced from the respondents’ comments included placing more emphasis on preventive care and the availability of non-pharmaceutical therapies and generic drugs to treat patients with rare diseases. Additional file 3 presents sample comments provided by the survey respondents for each theme.

## Discussion

Our survey results based on 1631 responses across Canada were that *Improved Quality of Life* and *Effective Health Care* were priorities most frequently selected among the survey respondents that should be considered when making decisions about funding of DRDs. Although reimbursement decisions are made by the provincial and territorial drug plans, a majority of respondents also agreed with the scenarios that all Canadians should have equal access to the same drugs and to fund a DRD only if it is justified in terms of the drug’s cost-effectiveness. As per their descriptions, both scenarios support the *Effective Health Care* and *Cost Containment* priorities (Table 1). Our analyses identified associations between the funding scenarios and several priority selections. Although access to DRDs varies by jurisdiction, we did not find a significant relationship between the agreement with the funding scenarios and geographic location. Regardless of the geographic location, a majority of the respondents support federal drug assessment that would determine which drugs are deemed as providing value for money for coverage, and that coverage should serve the goal of ensuring that Canadians have the same access to DRDs across all jurisdictions.

Our survey findings were aligned with the results of a 2014 study that presented 13 trade-off scenarios to 2111 participants to measure values of the Canadian public on drug prioritization and reimbursement for rare diseases versus common diseases and other non-health care programs [16]. More specifically, both studies found that participants supported national equity for DRDs [16].

### Limitations

The interpretation and application of our study may be influenced by several limitations. The survey presented a description of each priority and scenario, but it is uncertain how the respondents interpreted the descriptions and if they required additional information for clarification purposes. Moreover, they did not have much of an opportunity to consider expert and stakeholder input, and our methodology did not use time-trade-off required by decision makers when prioritizing or allocating finite resources. The survey responses suggested that a majority would support national consistency of funding decisions based on evidence of comparative cost-effectiveness, and 50% agree to fund DRDs. Although the survey responses highlighted a diversity of perspectives, it is not feasible to identify all Canadian perspectives based on the number of limited number of respondents [9, 11]. In the current sample, a greater proportion of our survey respondents had a college diploma or university degree or earned at least $100,000 compared with the Canadian population [17, 18]. Although the survey sampling targets were not based on the actual Canadian population density, targeted demographics by geographic area, as well as age and sex, were specified and were within 5% of the targets. This approach helped ensure that a variety of perspectives were represented for comparative purposes.

### Directions for future research

The survey responses will help to inform the planning and implementing a deliberative public engagement workshop on decision-making processes for DRDs. The theory of deliberative democracy argues that the mini-forums created for the deliberative engagement present advice that is the best estimate of how citizens would advise knowledge users if citizens were informed, civic-minded and deliberative [19]. It is a common belief that citizens are not generally well informed, nor are they supported to consider the perspectives of others and the common good. That said, the online survey provides an estimate of advice from non-deliberating informed citizens. The responses can be considered by the deliberation participants, and by knowledge users as they decide whether and how to incorporate advice from these differently constituted forums. One objective of this study was to use demographic associations, including geographical location, with different the survey responses to guide the sample selection for the deliberative engagement. Initial analyses did not identify any statistically significant associations that can be used for this purpose. However, themes that emerged from the content analysis can help to identify information that participants might require to support deliberation, which can be provided prior to the workshop and during the deliberations. For example, several comments were associated with the promotion of preventive care and use of generic drugs as a treatment option. A large proportion of rare diseases have genetic origins, and DRDs usually have extended patent protection compared with therapies for more common indications. The current survey uses the Likert scale to gauge the respondents’ top priorities and level of agreement with the funding scenarios associated with DRD funding. It remains uncertain if the results of this survey align with the perspectives of the decision-making bodies in Canada, especially in jurisdictions with programs for DRDs. An analysis on the Canadian public’s perspectives compared with the values and principles of these programs is merited. Studies on the public’s perspectives in other countries and how they contrast with those among Canadians are also warranted. Future research can apply the trade-off technique to measure the Canadian public’s perspectives in which respondents must consider and compare all key attributes associated with each priority and funding scenario. Their responses can then be compared with those derived from this survey.

## Conclusions

An online national survey was conducted to inquire about the Canadian publics’ priorities and perspectives on funding decisions for expensive drugs for rare diseases. Among the 1631 respondents, *Improved Quality of Life* and *Effective Health Care* were most frequently selected as the top priorities. Ensuring equal access across Canada and funding for DRD only when the drugs were deemed to be cost-effective had the highest agreement level among the survey respondents, while 50% agreed that DRDs should be funded independent of their effectiveness. Our analyses did not identify any significant relationships between the rating of the scenarios and demographics, including geographic location. The survey results suggest that while a policy that provided national equity for cost-effective drugs would be widely supported, there are likely to be a significant number of citizens who will object to any restrictions to access to DRDs.

## Additional files

Additional file 1: Screen Captures of Online National Survey. Supplementary file 1 presents the screen captures of the online national survey on drugs for rare diseases. (DOCX 1070 kb)

Additional file 2: Frequency of Priority Rankings by Province. Supplementary file 2 presents the results of the frequency of priority rankings by jurisdiction. (DOCX 26 kb)

Additional file 3: Comments from Survey Respondents. Supplementary file 3 presents a sample of the written comments from the survey respondents. (DOCX 55 kb)

## Abbreviations

CDN: Canadian
CIHR: Canadian Institutes of Health Research
DRDs: Drugs for rare diseases
MCDA: Multi-criteria decision analysis
