# Supplementary figures and images for: Engaging the Canadian public on reimbursement decision-making for drugs for rare diseases: a national online survey
Source: BMC Health Serv Res. 2017 May 26;17:372. doi: 10.1186/s12913-017-2310-4 (PMC5446683; doi:10.1186/s12913-017-2310-4)

**Supplementary File 1: Screen Captures of Online National Survey**


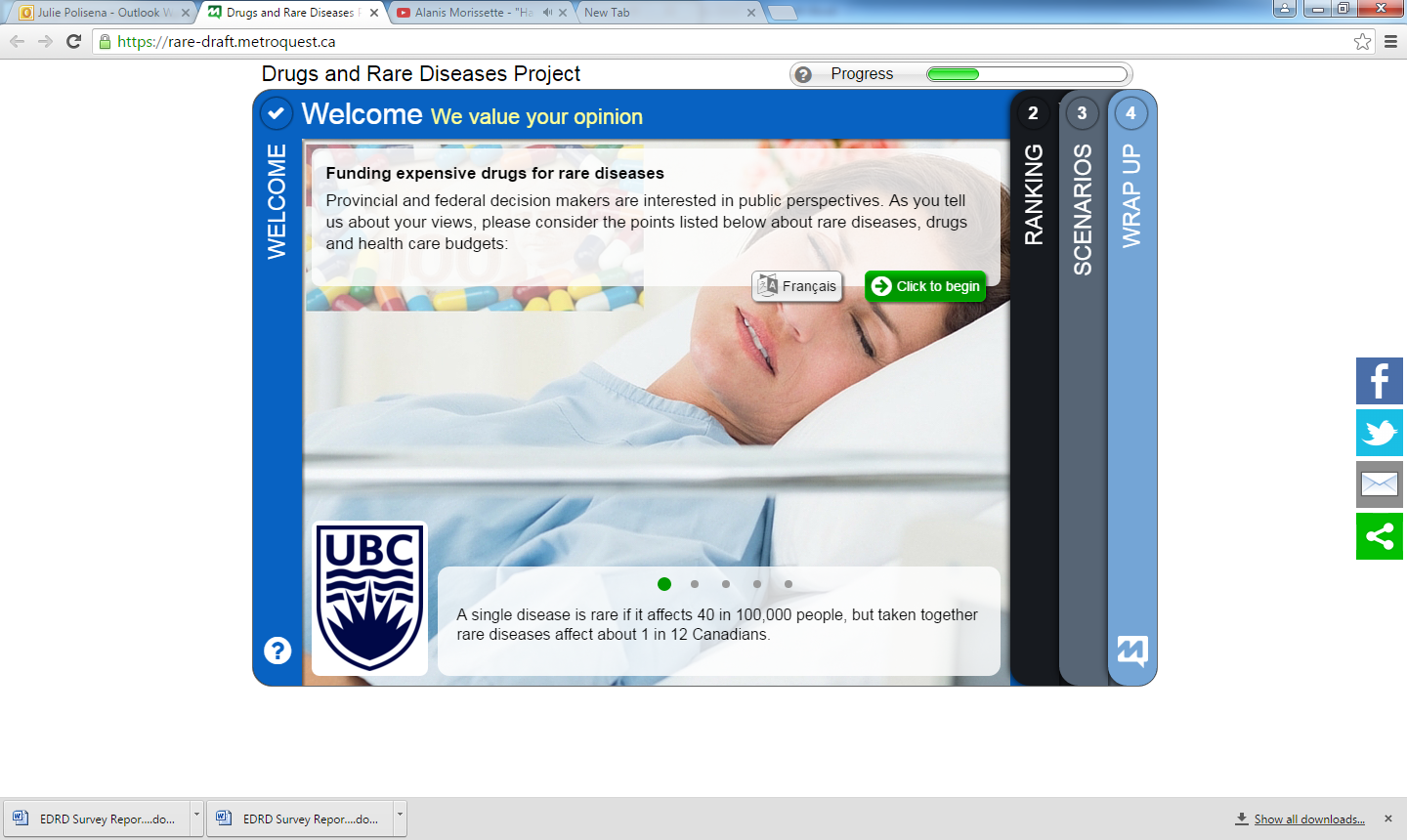


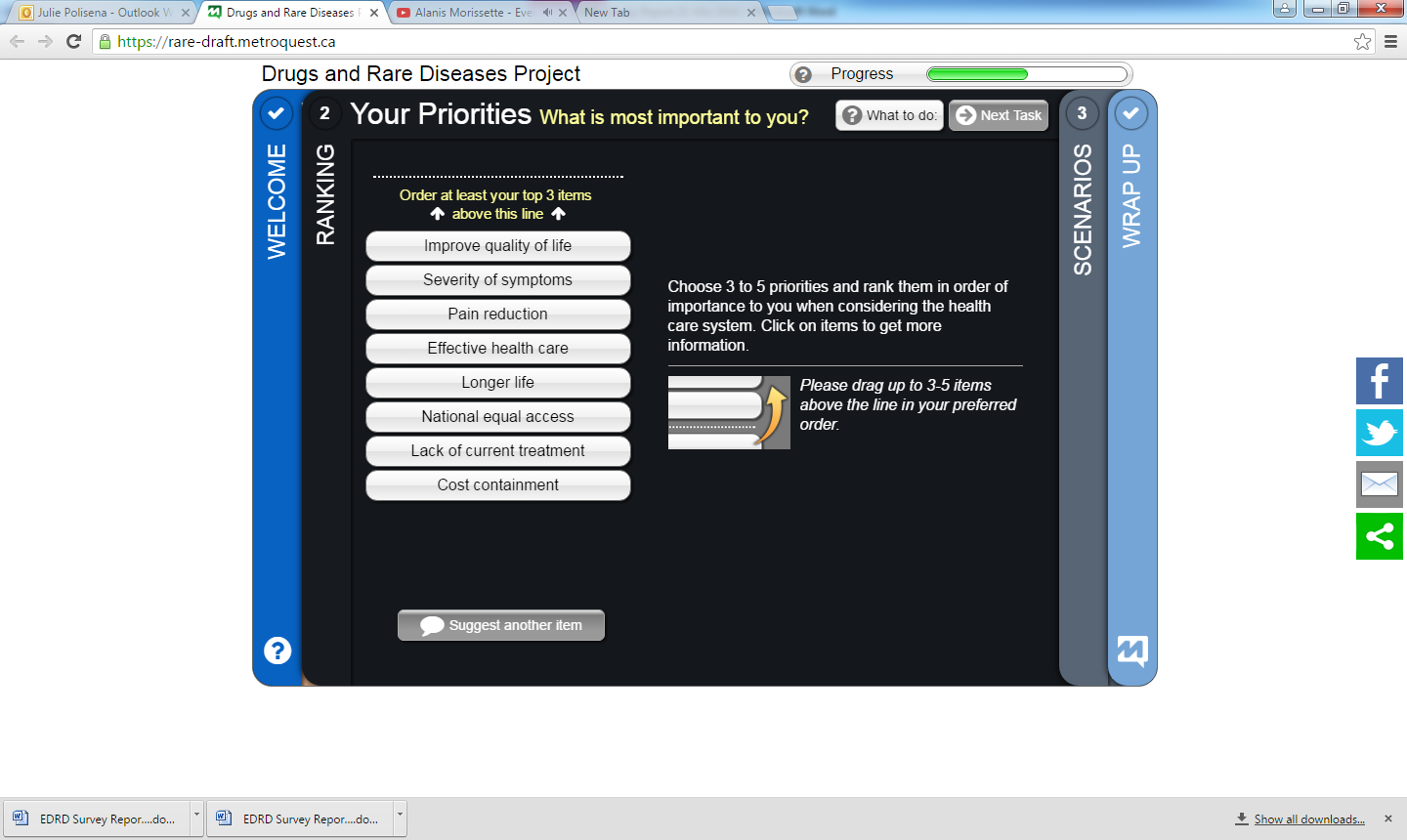


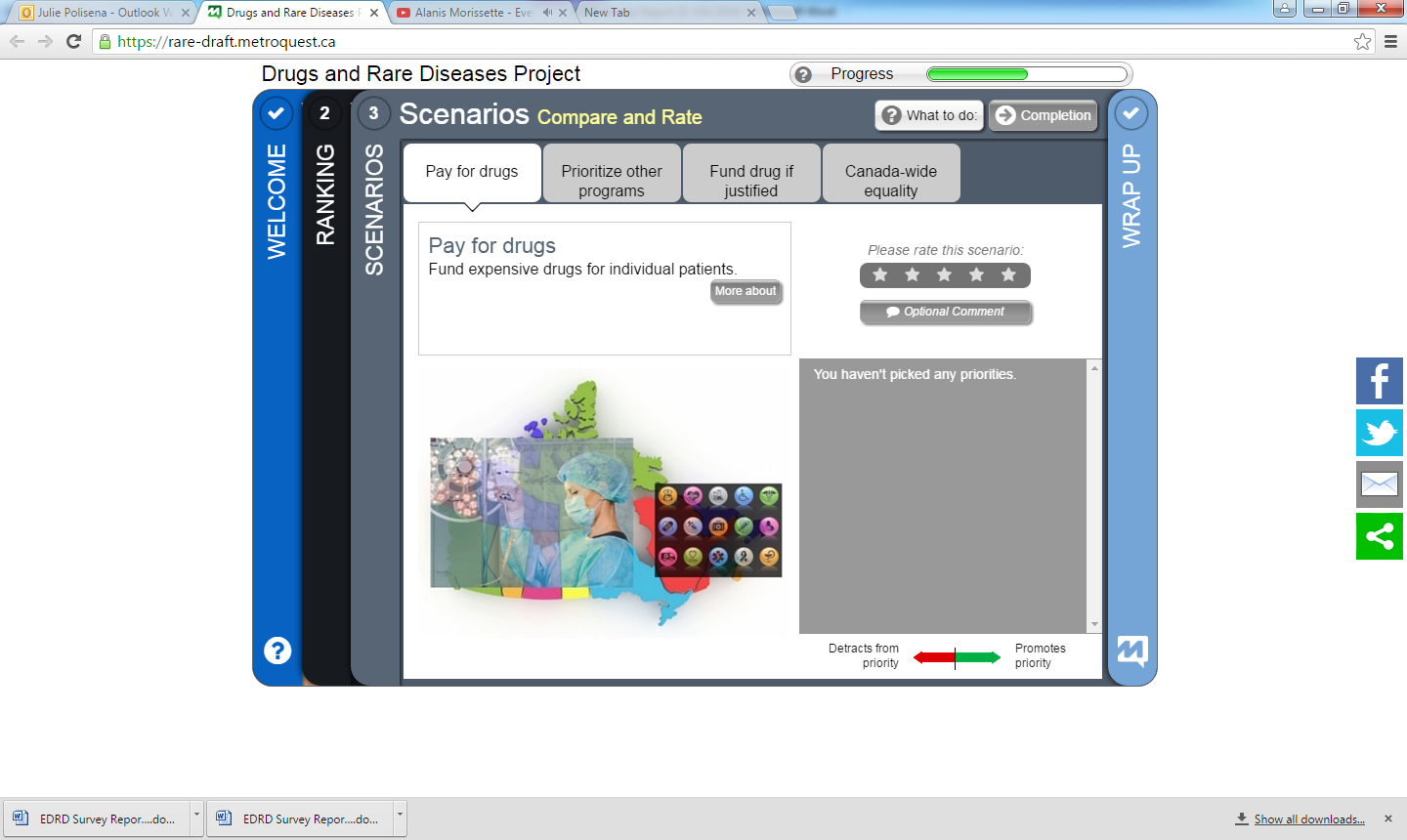


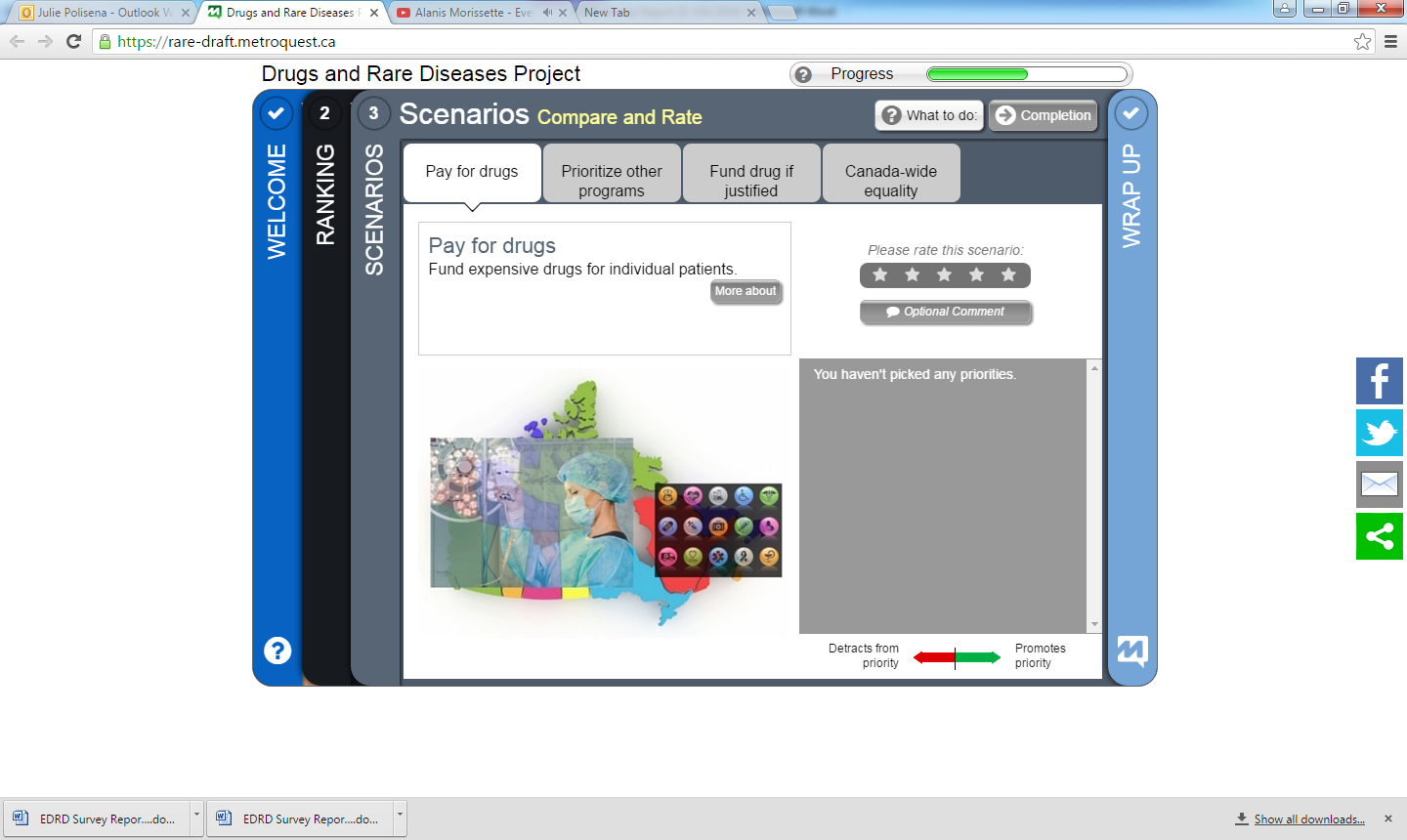

Supplement: Supplementary file 1 — Screen Captures of Online National Survey. Supplementary file 1 presents the screen captures of the online national survey on drugs for rare diseases. (DOCX 1070 kb) [file 12913_2017_2310_MOESM1_ESM.docx]
