# Supplementary material for: Engaging the Canadian public on reimbursement decision-making for drugs for rare diseases: a national online survey
Source: BMC Health Serv Res. 2017 May 26;17:372. doi: 10.1186/s12913-017-2310-4 (PMC5446683; doi:10.1186/s12913-017-2310-4)
Supplement: Supplementary file 2 — Frequency of Priority Rankings by Province. Supplementary file 2 presents the results of the frequency of priority rankings by jurisdiction. (DOCX 26 kb) [file 12913_2017_2310_MOESM2_ESM.docx]

**Supplementary File 2: Frequency of Priority Rankings by Province**

| **Priority** | **Ranking** | **Frequency** | | | | | | | | | | |
| --- | --- | --- | --- | --- | --- | --- | --- | --- | --- | --- | --- | --- |
|  |  | BC (n=138) | AB  (n=162) | SK  (n=145) | MB (n=157) | ON (n=130) | NCR (n=20) | QC (=130) | NS (n=55) | NB (n=42) | PEI (n=6) | NL (n=113) |
| Pain Reduction | 1 | 6 | 11 | 12 | 10 | 7 | 2 | 11 | 2 | 4 | 1 | 2 |
|  | 2 | 15 | 9 | 19 | 18 | 11 | 2 | 32 | 7 | 4 | 0 | 16 |
|  | 3 | 18 | 20 | 19 | 11 | 13 | 4 | 23 | 4 | 5 | 1 | 10 |
|  | 4 | 5 | 9 | 5 | 15 | 5 | 2 | 3 | 4 | 1 | 0 | 8 |
|  | 5 | 1 | 9 | 8 | 2 | 6 | 0 | 3 | 1 | 1 | 0 | 3 |
|  | No response | 93 | 104 | 82 | 101 | 88 | 10 | 58 | 37 | 27 | 4 | 72 |
| Improve Quality of Life | 1 | 37 | 40 | 33 | 44 | 44 | 8 | 43 | 21 | 17 | 1 | 36 |
|  | 2 | 42 | 57 | 40 | 43 | 29 | 4 | 39 | 9 | 7 | 1 | 32 |
|  | 3 | 26 | 31 | 37 | 29 | 27 | 0 | 20 | 12 | 9 | 1 | 22 |
|  | 4 | 5 | 3 | 6 | 6 | 3 | 0 | 7 | 1 | 0 | 0 | 3 |
|  | 5 | 1 | 0 | 1 | 4 | 1 | 2 | 1 | 3 | 0 | 0 | 2 |
|  | No response | 27 | 31 | 28 | 31 | 26 | 6 | 20 | 9 | 9 | 3 | 18 |
| Longer Life | 1 | 9 | 11 | 5 | 16 | 4 | 1 | 1 | 3 | 6 | 2 | 7 |
|  | 2 | 7 | 9 | 12 | 8 | 14 | 1 | 3 | 4 | 2 | 0 | 10 |
|  | 3 | 18 | 12 | 7 | 14 | 7 | 2 | 7 | 3 | 4 | 0 | 11 |
|  | 4 | 2 | 2 | 1 | 2 | 2 | 0 | 2 | 1 | 1 | 0 | 1 |
|  | 5 | 1 | 3 | 2 | 4 | 1 | 0 | 2 | 1 | 0 | 0 | 3 |
|  | No response | 101 | 125 | 118 | 113 | 102 | 16 | 115 | 43 | 29 | 4 | 81 |
| National Equal Access | 1 | 18 | 23 | 15 | 17 | 13 | 2 | 15 | 14 | 7 | 0 | 14 |
|  | 2 | 17 | 15 | 13 | 15 | 13 | 1 | 11 | 7 | 9 | 2 | 10 |
|  | 3 | 13 | 19 | 18 | 23 | 20 | 1 | 19 | 9 | 2 | 0 | 15 |
|  | 4 | 7 | 12 | 2 | 9 | 3 | 1 | 2 | 1 | 3 | 0 | 7 |
|  | 5 | 4 | 0 | 6 | 5 | 4 | 0 | 4 | 1 | 3 | 0 | 3 |
|  | No response | 79 | 93 | 91 | 88 | 77 | 15 | 79 | 23 | 18 | 4 | 64 |
| Lack of Current Treatment | 1 | 8 | 4 | 5 | 8 | 10 | 0 | 5 | 1 | 1 | 0 | 7 |
|  | 2 | 7 | 10 | 14 | 16 | 5 | 0 | 6 | 4 | 0 | 0 | 10 |
|  | 3 | 13 | 12 | 10 | 10 | 9 | 1 | 8 | 4 | 5 | 1 | 4 |
|  | 4 | 2 | 2 | 4 | 4 | 6 | 1 | 1 | 3 | 3 | 0 | 3 |
|  | 5 | 4 | 3 | 0 | 1 | 1 | 0 | 2 | 0 | 2 | 0 | 3 |
|  | No response | 104 | 131 | 112 | 118 | 99 | 18 | 106 | 43 | 31 | 5 | 86 |
| Severity of Symptoms | 1 | 8 | 5 | 5 | 14 | 2 | 1 | 5 | 3 | 0 | 0 | 6 |
|  | 2 | 12 | 13 | 9 | 11 | 16 | 1 | 5 | 8 | 1 | 0 | 5 |
|  | 3 | 12 | 17 | 11 | 21 | 11 | 4 | 6 | 8 | 2 | 0 | 11 |
|  | 4 | 5 | 4 | 6 | 4 | 7 | 1 | 6 | 2 | 2 | 0 | 5 |
|  | 5 | 2 | 5 | 1 | 4 | 1 | 0 | 4 | 0 | 0 | 0 | 2 |
|  | No response | 102 | 115 | 113 | 103 | 93 | 13 | 104 | 34 | 37 | 6 | 84 |
| Cost Containment | 1 | 8 | 5 | 11 | 1 | 6 | 1 | 7 | 0 | 0 | 0 | 1 |
|  | 2 | 15 | 13 | 12 | 8 | 8 | 3 | 7 | 2 | 6 | 2 | 9 |
|  | 3 | 15 | 25 | 15 | 23 | 22 | 4 | 19 | 8 | 5 | 2 | 12 |
|  | 4 | 4 | 6 | 8 | 8 | 5 | 0 | 4 | 1 | 1 | 0 | 8 |
|  | 5 | 5 | 6 | 2 | 6 | 1 | 1 | 7 | 2 | 1 | 0 | 2 |
|  | No response | 91 | 107 | 97 | 111 | 88 | 11 | 86 | 42 | 29 | 1 | 76 |
| Effective Health Care | 1 | 47 | 60 | 59 | 47 | 44 | 5 | 41 | 11 | 7 | 1 | 33 |
|  | 2 | 23 | 36 | 26 | 38 | 34 | 8 | 27 | 14 | 13 | 1 | 21 |
|  | 3 | 23 | 26 | 28 | 26 | 21 | 4 | 28 | 7 | 10 | 1 | 28 |
|  | 4 | 1 | 2 | 5 | 2 | 0 | 0 | 3 | 3 | 1 | 0 | 1 |
|  | 5 | 2 | 1 | 1 | 3 | 3 | 1 | 1 | 0 | 0 | 0 | 0 |
|  | No response | 42 | 37 | 26 | 41 | 28 | 2 | 30 | 20 | 11 | 3 | 26 |

**Supplementary File 2: Frequency of Rating of Scenarios by Province**

| **Description** | **Rating (1=lowest and 5 highest)** | **Frequency** | | | | | | | | | | |
| --- | --- | --- | --- | --- | --- | --- | --- | --- | --- | --- | --- | --- |
|  |  | BC (n=138) | AB  (n=162) | SK  (n=145) | MB (n=157) | ON (n=130) | NCR (n=20) | QC (=130) | NS (n=55) | NB (n=42) | PEI (n=6) | NL (n=113) |
| Fund Drug if Justified | 1 | 1 | 6 | 0 | 4 | 2 | 2 | 4 | 3 | 0 | 0 | 1 |
|  | 2 | 7 | 10 | 7 | 10 | 2 | 1 | 5 | 3 | 2 | 0 | 5 |
|  | 3 | 37 | 26 | 29 | 33 | 25 | 1 | 20 | 11 | 11 | 4 | 15 |
|  | 4 | 38 | 66 | 62 | 57 | 50 | 7 | 56 | 13 | 13 | 1 | 46 |
|  | 5 | 52 | 53 | 45 | 51 | 51 | 8 | 44 | 25 | 16 | 1 | 46 |
|  | No response | 3 | 1 | 2 | 2 | 0 | 1 | 1 | 0 | 0 | 0 | 0 |
| Canadawide Equality | 1 | 4 | 8 | 1 | 3 | 6 | 1 | 1 | 0 | 0 | 0 | 1 |
|  | 2 | 2 | 9 | 5 | 8 | 2 | 0 | 7 | 1 | 1 | 1 | 4 |
|  | 3 | 28 | 30 | 16 | 21 | 20 | 3 | 16 | 9 | 5 | 2 | 10 |
|  | 4 | 38 | 36 | 36 | 48 | 42 | 2 | 39 | 16 | 9 | 1 | 29 |
|  | 5 | 65 | 76 | 86 | 76 | 60 | 13 | 66 | 29 | 27 | 2 | 69 |
|  | No response | 1 | 3 | 1 | 1 | 0 | 1 | 1 | 0 | 0 | 0 | 0 |
| Prioritize Other Programs | 1 | 13 | 13 | 10 | 16 | 12 | 1 | 17 | 4 | 4 | 1 | 9 |
|  | 2 | 19 | 32 | 28 | 32 | 21 | 2 | 31 | 15 | 9 | 1 | 22 |
|  | 3 | 47 | 46 | 49 | 51 | 50 | 9 | 39 | 18 | 10 | 1 | 42 |
|  | 4 | 37 | 54 | 35 | 39 | 28 | 5 | 29 | 12 | 11 | 2 | 29 |
|  | 5 | 19 | 15 | 21 | 17 | 18 | 0 | 13 | 6 | 7 | 1 | 11 |
|  | No response | 3 | 2 | 2 | 2 | 1 | 2 | 1 | 0 | 1 | 0 | 0 |
| Pay for Drugs | 1 | 6 | 14 | 6 | 6 | 9 | 2 | 8 | 4 | 1 | 0 | 5 |
|  | 2 | 20 | 32 | 20 | 24 | 18 | 3 | 13 | 13 | 4 | 2 | 13 |
|  | 3 | 45 | 45 | 45 | 49 | 37 | 5 | 37 | 17 | 12 | 2 | 39 |
|  | 4 | 39 | 42 | 40 | 45 | 40 | 5 | 45 | 13 | 16 | 1 | 27 |
|  | 5 | 23 | 27 | 33 | 32 | 24 | 4 | 24 | 7 | 9 | 1 | 29 |
|  | No response | 5 | 2 | 1 | 1 | 2 | 1 | 3 | 1 | 0 | 0 | 0 |
