# Supplementary material for: Engaging the Canadian public on reimbursement decision-making for drugs for rare diseases: a national online survey
Source: BMC Health Serv Res. 2017 May 26;17:372. doi: 10.1186/s12913-017-2310-4 (PMC5446683; doi:10.1186/s12913-017-2310-4)
Supplement: Supplementary file 3 — Comments from Survey Respondents. Supplementary file 3 presents a sample of the written comments from the survey respondents. (DOCX 55 kb) [file 12913_2017_2310_MOESM3_ESM.docx]

**Supplementary File 3: Comments from Survey Respondents**

| **Is there anything else it is important for us to consider?** |
| --- |
| efficiency is a must |
| Coverage under Provincial plans as opposed to private plans. |
| everyone should be able to have access to help with expensive drugs.. |
| I believe it important for all Canadian to have access to medical assessment and treatment , but unfortunately those with rare and thus more expensive treatments are sometimes left behind , while others with very sublte or minimal disease utilise or abuse alot of their health care costs |
| Sharing the cost across all Canadians |
| Consider that actual effectiveness of the drug before funding an expensive drug. Hopefully consideration is given to the possibility of a generic branding instead. |
| geography, means and attitude |
| Use of expensive drugs to provide QUALITY longer life. |
| if it is for a child it matters more as they have a full life ahead of them |
| If not funded it places big financial burden on families |
| yes, everyone deserves to be able to afford their medication, even if you have to take the funds from other sources, drug company's shouldn't be making such high profits. |
| Expensive drugs should have funding from govt. |
| To me, the medical 'system' of each country seems quite insular - meaning procedures, systems, approaches. To answer the questions posed in this survey, as well as other ones, please consult internationally for solutions. |
| Treat every case as it's own problem. What works for one might not be what works for most. Every life is important and should be treated as such. |
| Those who can't afford it & require it. |
| It is important that people not have to go in debt because they cannot afford to purchase the drugs needed to improve their quality of life. |
| It appears as though different patients receive different levels of coverage even though they may fall into the same categories of pain, severity of illness, etc. |
| less expensive generic versions of drugs |
| financial burden to individuals is key - e.g., why should someone be put through undo hardships if they cannot afford drugs that will help their condition. |
| private funding raising together with government funding to ensure resources available; regularly reassess prices of drugs, if it may be overpriced, fair consideration to drug investors and tax payer equal |
| People having extended health care coverage from employers should have rare disease drugs covered there first - how to ensure those insurance companies will do their share to cover those employees and leave more government-funding available to those with no employer extended health coverage? |
| cost |
| Institute government regulation on maximum profit drug companies can make or they forfeit their copyright. Opt out of TPP, etc al. |
| Wait times to receive diagnosis, care and treatment |
| human life is more important than cost and money!! |
| While it is nice to have solutions for rare diseases since they affect so few I would say pain reduction and quality of life is more important than cures. |
| The government should look at funding for patients that need expensive drugs to sustain a good quality of life. |
| Drug research is important however should watch the budget on the matter as to control the national budget. |
| people with low income really cannot pay for drugs. Helping those in need for money to buy them drugs is very important. |
| While it is admirable to want to treat every Canadian's ailment no matter what the cost we have to realize that we have a limited amount of resources. We have to use those resources as responsibly as possible at that means trying to help the most people as possible with those limited resources. Unfortunately, that also means not being able to help every person in every possible scenario. We have to ensure the majority of treatments are both cost effective and offer tangible results. |
| Quality of life for our elders in care facilities is not what it should be. Elderly are not being looked after to the best potential |
| Funding should be a priority if there are no other alternatives and the drug must be effective for the patient |
| to much more of low income people for some drugs |
| Give equal considerations to all views/opinions and do not cherry pick facts that support your viewpoint. |
| Mostly covered by private insurance |
| Having input from the physician to justify the need for the expensive drug to receive approval. |
| Funding of this nature should only apply to the working citizens of this country and not the useless welfare soaking bums who can work but choose not to. I still feel sick to the stomach seeing the druggies and winos around the liquor store all get welfare checks and free healthcare (OHIP) that my hard earned dollars are paying for. |
| Hybrid healthcare system is needed, the original vision of universal health care can no longer be sustained with chronic strains on the health system ie. people visiting emergency rooms for colds and flu those who abuse the system should be billed |
| I already mentioned it, but I will repeat it here. In the case of rare diseases, the development of drug treatments may be prohibitively expensive. However, if patients are allowed to make their own decisions about their health, allow them to get together and try remedies which are not available through the public health system. I am not familiar with all the details of how new drugs are approved, but I would guess that much of the prohibitive cost would be due to the regulative process? |
| Because health care is government funded the quality of the doctors, dentists, opticians, GPs is poor almost lacking in knowledge and medical rigour. Improve the quality of these medical practitioners by having exams each year to test their knowledge and skills. |
| how long a wait for tests should be concerned. |
| Every life is valuable. If there is something that can be done to save a life, or to make whatever remains of their life more comfortable and enjoyable, then cost should not be a factor, and should not be a worry for the families. |
| Just pointing out no one care until it is there loved on that is effective everyone is equal and should be treated as such and everyone should have access across Canada equally and we should come up with alternative and more natural cheaper alternatives if possible we do not need to make the drug companies richer |
| the wealth status of the patient. if they are rich, let them buy their own medicines |
| It is difficult to say that one deserves more coverage than another person. It would be great if the country could come together and assess the individual needs of the person in front of you without any bias. |
| Makes most sense to find cures to diseases that affect the most of general population |
| doctors need to look at what you have been though, the effect of trama, and life events and not round peg round whole everyone. someone who survives an assault is left disabled, and with related diseases from those events an is in pain to the point of vomiting should have access to pain meds that work for them and shouldn't be punished anymore because of others, or the dislike of government or doctors they should also be looked at differently when deciding what numbers they should be between someone with rare diseases don't need the added stress of having to fit in those boxes, blood pressure, blood sugar, i deal with two people with disease that cause pain, we're told the pain raises numbers but they are expected to keep the numbers in the norm then the doctors focus becomes those numbers instead of the pain related disease making them suffer when if they treated the disease with what works for that person the numbers should come closer to norm but will never be perfect |
| it shouldn't matter what the illness, but the government needs to crack down on the drug companies ripping off everyone with outrageous costs |
| IF THE DIESEASE IS VERY PAINFULL FOR AN INDIVIDUAL AND HARD TO LOOK AFTER THE PATIENT FOR THEIR CARETAKERS AND IF TSAID PATIENT IS GOING DIE AT THE END, THE DECISION SHOULD BE MADE BY THE FAMILY TO END THE LIFE. |
| One could someone speed up the process |
| we need to be prioritize funding but also be realistic |
| There shouldn't be anything to think about. Drug companies are out to make billions and only worry about the people that can afford their medications. Everyone should be treated equal and on the same playing field. |
| costs have to be weighed but so does long term expectations, ie does this person expect to live a long life with this drug? If not, even less finding should be provided |
| Quality of global life, if its a coordinated global effort will be more effective |
| Many people who need expensive drugs cannot afford them. As a socialist country, it is important that the gov't fund these drugs, no matter what the disease. |
| This country foolishly spends money. We let corrupt big business get away with murder for how they do not pay taxes, create unsafe work enviornment and destroy the planet. This country also spends way to much on military and security. IF money was budgeted better we could do even more than have a better medicare system. |
| Must be analyzed holistically. Can't look at cost, or incidence of the disease or any factor in isolation. Decisions about funding must be made looking at all factors globally |
| The challenge in this area is finding the balance between insuring all patients with or without rare disease receive treatment and balancing that with the cost to the tax payers. Where that balance is, is hard to determine - particularly when individual stories are heard about in the news as people challenge to get the treatment they think they need |
| its hard for parents of a very sick child to be working double time to pay for medications,and never be able to see your own child because your just trying to keep him or her alive.these people need help. |
| I think more research should go into finding affordable meds |
| These cases should not have to be written about in the media for the government to take action and then re-action when their is a public outcry. Make the right decision in the first place. People's emotions are affected as much as one's physical ailments. |
| Not everyone has access to benefits through their employer. Even when reasonably employed, some drugs cause monetary distress. |
| Don't fund pseudodrugs that haven't been clinically proven to be effective. |
| none other then to have ways for low income falimys to help pay them full or half of it. |
| Government funding vs. insurance coverage |
| I often hear the drug companies' arguments that Canada is a wealthier country than those on the African subcontinent etc. When dealing with the costs of rare diseases, the patients' own income should the definitive factor, not some ultra-rich company's opinion of the landmass they reside on. The Canadian government allows businesses to tell it if its population deserve access to treatment? For shame. |
| percentage of canadians needing them; taking note of whether people have caused their own illness, like smoking, drinking, and work on how those things can be treated first to allow for prevention; letting the drug trials be possible for all people who need treatment, not just people who have the money; educate people about diseases and what is truly available |
| Perhaps if provincial tax budgets are not wasted on patronage programs, rich & corporate welfare and a whole host of other non-necessities then allocating the funds for this kind of drug planning would be easy. Keep the infrastructure in good shape, operate the schools and make sure medicare is well funded works....that's all we really ask....not helping your buddy's business out, or thinking that economic stimulus comes from corporate tax breaks. |
| Drug companies make a LOT of money, and government must be careful they don't get swindled or taken advantage of. Drug companies seem to inflate their prices with weak justification. |
| Consider all aspects- cost, other options, ability of patient to pay, possible outcome |
| Fight the root problem which is the expense of the drug. Fund in country research and development of treatment that will help Canadians. |
| people should not need to choose between paying for drugs for their health and paying for food. Expensive ones should be funded, especially if its a daily medication. |
| Have doctor's take telephone calls with patients for quick things such as prescription refills, etc. rather than having to book an appt. Also, make all test results available online so we don't have to come in for our results. |
| Please make generic versions available, and they should have priority |
| People without good healthcare plans through their work should be allowed equal access as those that have good plans through their workplace. |
| Difficult subject. I believe that by charging users a fee for non emergency items or optional treatments we could divert a considerable amount of funds from the system that could fund items that the average person has no chance to fund themselves. |
| Find out why they are expensive and maybe look for grant or other private solutions. |
| The way the families of those with rare conditions go to the media, making sure the provincial governments have the ability to rationalize their response to the case to provide a balanced story to the media. |
| Our son has PKU and the drug KUVAN that is new for use with PKU is extremely expensive and as PKU is a rare disease it would be amazing if it were covered, or even partly. |
| SHOULD BE EQUAL FOR EVERYONE AS LONG AS THEY NEED THEM NOT FOR ABUSE |
| Healthcare should be equal for all of us but should be closely regulated to avoid abuse of the system. |
| Cost controls to keep drug prices down. |
| is this person being kept alive when we should be letting them go (i.e. terminal patient that will have no quality of life with the drugs) |
| We live in Canada so medical care should be covered. |
| Drug companies are seeking to recoup all development costs and maybe if more money was put into development, the cost would not be so high |
| There are no easy answers. I do not believe that everything should be covered every time but I do believe as a public policy position there should be no differentiation across the country to a drug, only that access criteria are well-established and fair. And decisions should be based on real data, not emotions or over-the-top media coverage. Fairness is paramount and that will not mean equal access if the criteria are not met. |
| I support a Canada wide drug purchasing scheme where bulk purchase can/should reduce cost. |
| Advocating for additional therapies that could provide relief and not jeopardize the famil members assisting - i.e. legalize marijuana for medical use and allow patients to be able to legally provide for their own private use |
| I am a volunteer with Cystic Fibrosis Canada and have been involved with Provincial advocacy. In the case of CF the new drugs may allow CF individuals to live longer and be productive members of society rather than dependent on. |
| There should be more information on generic drugs and are they really as good as the original. |
| Fund ALL drugs |
| Try to avoid being swayed by pressure from journalists, particularly regarding individual cases, or high profile individual cases. |
| Drug decisions should be addressed by doctors as well as politicans |
| Pharmaceutical companies should bear an ethical and financial responsibility for the development of these drugs given the astronomical profits that make |
| It may be important for funding accountability to have the reasoning behind why certain drugs are so expensive - Fund the solutions to make them less expensive (is it something we can grow/harvest in our own country? |
| funding of expensive drugs should be backed by research information data that is not biased based on who provided funding for the research itself. |
| The problem with making funding decision is the "red tape" and the lack of organizations to communicate effectively with each other. Sometimes the time lapses are huge and then the treatments are not as effective. |
| There is a two tier system, those that can't afford medication like welfare recipients get the generic brand of drugs. Those if their own health care plans can up it a notch. I question really is there a difference in the quality of the medication, are they equally effective. That being the case why not generic across the board. Pharmaceutical companies companies need to be reined in. |
| impact on the funding of health care for the majority of Canadians |
| Monitor pharmaceutical companies |
| A consideration should be the age of the patient. The sooner a diagnosis is made the better chance a patient has of seeing improvement because of expensive drugs. There has to be some type of return on investment for both the patient and the health care system |
| Consider financial situation of the recipient. |
| This study seems to be geared towards cost containment rather than right to treatment. Although I favour right to die legislation I wonder what the bean counters in government would do if this was more "cost effective" than treatment. |
| I'm not sure there is a correct solution unless you add unlimited funding. It's always going to come down to which side of the fence you are on. Never going to be an easy decision when money is also involved. |
| My husband has multiple sclerosis so I totally understand about us having the support of the Federal and Provincial health care. |
| By definition, a rare disease does not affect many people. The cost of treatments for such afflictions should not be excessive unless there is hope for curing the illness. |
| The cost of some essential life saving drugs is of great concern. |
| The effectiveness of the drug needs to be considered. |
| We pay high taxes for health care and over the years that health care is more expensive and less effective. Need to fix the system which may not be throwing money at it. |
| who puts the price tag on the drugs.....pharmaceutical companies that want to be paid for their research. But sometimes they have had tax breaks from the government, incentives to locate their facilities in Quebec for example and have already been recompensed for some costs. One sees such greed in the sector it's hard to justify the costs of some medications, when you know there is a patent for 35 years on a drug and they will recoup the costs very early in that time frame. Shouldn't the costs be amortized over 5-10 years, just like the research into the drugs? |
| The scenarios given do not consider anything in the area of alternatives to drugs, but perhaps that is a topic for future consideration. |
| Expensive drugs should be funded for a set period of time. |
| Diagnostic tests for such things as cancer should be free and done at frquent intervals. Early diagnosis would reduce costs involved in late stage diagnosis |
| All drugs, including expensive ones, should be funded; however, the priority should be on increasing a healthy lifestyle to reduce the need for drugs. The quality and comfort of every ones life, including those with rare diseases, should be a priority. |
| Even though we are not all ailed with desease, as long as everyone has equal access to a good heathcare system that is effective at prescribing safe drugs for the individual is the right step. I am happy if my tax funds go towards the betterment of someone who has a disease that lowers their quality of life because we live in a free and open society. If they aren't able to have a good day, but I am how is that fair? |
| Makes sense to subsidize preventive measures (quitting smoking drugs etc.) to avoid higher costs later on. |
| It is difficult for the public to accept that a drug funded in one province is not funded elsewhere. So there should be some attempt made by the provinces and the federal government to support some kind of equality of provision. We do need to contain public expectation though and that is the most difficult part - expensive drugs or access to emergency care or whatever the issue is - we can't have it all everywhere and more emphasis on preventive health, community clinics with nurse practitioners and supportive services need to be more available in rural regions to assist in the general health field. If we can find savings by keeping people out of hospitals and not doing expensive medical procedures for every ache and pain, then maybe we can provide suitable and effective care - like expensive drugs - when needed. It is very difficult to make intelligent comments when we only have some of the information. Health care is only one of a range of needs in society and expensive drugs only one piece of a much larger health puzzle... |
| Legislation to require drug companies to charge less?? is it possible? They seem to have too much control. |
| Apply means test to determine percentage of drugs to be funded versus individual's responsibility. |
| Canada Wide hard bargaining with drug companies with large volume discounts |
| Just because someone has a rare disease doesn't mean that their life is less valuable to fund. |
| Alternate medicine or methods should be considered reasonable if patient is approved for expensive drugs. That is, if approved for use of expensive drug (eg. $100/pill) then if they choose to use alternative healing the funding up to the cost of the medication should be covered. |
| Prevention is key. For ex: start promoting plant-based diets and healthy exercise suggestions as prevention from many diseases and conditions. Prevention is the key and will save unbelievably!! |
| In the end, its subjective. If It were my child, my spouse, my responses would be very different. This is a very abstract response. |
| Remember that not all Canadians are RICH and so quality drugs should be accessible for all. |
| Quality of life issues need to be at the forefront. Don't prolong the inevitable. |
| I am concerned that some drugs, not necessarily the most expensive, which seem to be working well, are suddenly removed from availability. |
| Income of patient Life expectancy |
| No clue why people have to stress out so much for expensive medications to keep themselves or their children alive! |
| Generics are ok |
| would death be eminent and would the drug be of significant help? |
| Cost is key....I'm all for using drugs that are cost effective, but as a taxpayer I don't want to publicly fund highly expensive drug treatments. I'm not against a two tier system whereby those who can afford it are allowed to choose to spend their own money on expensive drug treatments that are not publicly available to all people. |
| people that have lived in canada their whole lives should be funded for complete health care those just coming to the country that have never put anything into the economics should not be fully funded sad but people must put something into the country before receiving something from the country |
| cost shouldn't matter in saving lives |
| Governments rarely are "players" in new drug production--rather they are at the "mercy" of large drug companies who are essentially profit driven. Governments "investing" in drug production companies may be a way to improve returns to their taxpayers as well as those with complex and expensive drug needs. |
| If it is your life or a loved ones, how can a person NOT have funding regarding expensive drugs. |
| Drugs targeting younger population should take priority...i.e. children |
| Some other countries already have programs in place in which prescriptions drugs are paid for. If these programs also include coverage for drugs to treat rare diseases, those programs should be reviewed to see if their policies and procedures could be applied to a Canadian model. |
| reinforce my belief that all Canadians should be treated equally when it comes to health care. |
| Drugs are usually "cheaper" than hospital stays |
| we call Canada a free and democratic country which is equal and fair to all yet depending on where you live or your financial standing or even when you are a sitting member of a legislature or parliament people on what is loosely called a lower social standing either by demographic or finances get pushed aside by the above mentioned or even forgotten about talk is cheap action speaks volumes. |
| Yes as I personally suffer from chronic pain syndrome due to legge perthes disease as well as ankylosing spondylitis, as well as two advanced forms of arthritis hence I find the cost for generic narcotics are much less costly then say purdue-freferik morphine sr.200 mg, as well as statex morphine 25 mg., liquid methafone for this pain but definitely notice a difference between say the novopharm m.s.contin vs.purdue frederik m.s.contin 200 mg!!!???? |
| The improvement in the person's quality of life |
| National policy to reduce cost |
| Cost of drugs and complete checks on effectiveness. |
| there are medications that I take that don't work as well as others but have to take because they are not approved by pharmacare and can not afford. to me this is rediculus. I have to live in pain because the government won't fund these drugs. |
| National drug program is needed |
| Expensive drugs should be used judiciously. There must be clear cut protocols for their use across provinces or nationally.They cannot be at the whims of doctors. Expensive drugs must not be used in stuations where there is virtually a poor outcome guaranteed |
| Its all about helping people live a long and healthy life. |
| Public education. We also need to think about future cures. R&D costs money. That is why drugs can be expensive |
| reality |
| New and expensive drugs must be monitored when introduced. Side effects and progress must be documented to ensure these drugs are effective, with minimal side effects. |
| A family member has a rare disease and her onging treatment would not be possible if medications were not provided at an affordable price. Her quality of life is greatly enhanced by these medications. |
| The type of drug and is it essential |
| I do not feel I have enough knowledge or information on this subject to comment.  Socialized medicine has it's limits and always will. |
| I believe if a person's finance is checked by their income tax prior year and there is no way they can afford the expensive drug that will make their quality of life much better then they should qualify for government assistance. |
| I think it is important to take personal income into account. (BTW, I Have a very rare disease myself - Dercum's Disease) Some people are very wealthy and can easily pay for their own drugs no matter the expense. If that is the case, then allow them to do so easing the burden on the provinces. I know the issue will become the income cut-off but it would have to be realistic. Only the truly wealthy could actually afford most of these drugs. Most middle income families with minor children would be financially destroyed bearing the burden themselves. This is just a thought be it would seem ridiculous to me if wealthy people who could pay for their own drugs without it hurting their bottom line, had it funded. |
| I think that money should also be spent on researching alternatives to these very expensive drugs and quit pandering to the big drug companies. If they didn't charge such huge prices, they wouldn't be making the huge profits they make! How about serving the interests of humanity instead of a few peoples wallets! |
| Keeping Canada’s health care system strong, creating jobs and keeping communities safe are issues of top importance to Canadians. |
| A balance between care for a few and care for the majority. |
| Funding research so that gov't is a part owner of drug and can acquire it at a lower cost. |
| Some drugs need to be covered tha the seniors are dependant on |
| If there is Any Hope What so ever that the Drug could help a person they should have Full Paid Access to it with no money out of the patients pocket. |
| should be subsidized for all |
| Such a complex issue. Ideally all have access to treatment that improves quality of life, however that needs to be weighted on the impact of the broader system. |
| Cost is a factor but care of the sick is more important, waste of assets should be reduced through streamlining and standardizing treatments. |
| I am aware there is need for expensive drugs and I am aware that drug companies need to be profitable, the costs of very expensive drugs needs to be regulated. There should be more accountability on the part of drug companies to justify when a very expensive drug is approved for use. Research and development is obviously expensive and if it is for a drug that helps a small number of people than it is understandable that prices need to be high to cover expenses and make a profit, but a government regulated body or independent organization should be looking at the costs for both manufacturer and consumer and determining a fair market value to enable the drug companies to make a profit and at the same time not desicrate families. |
| Consider the individual's ability to pay for the drug themselves without they or their family being greatly impacted financially |
| I would think that for example: our work benefits cover a lot of my husband's expensive drugs that the government benefits/funding should go to other less fortunate individuals |
| Is the treatment available outside Canada? If so collaborate with other countries to provide the care needed. |
| Evaluation of drug company pricing strategies should be under review by gov't. If they get any public funding for research they should not be allowed to set high pricing for drugs. |
| Health and education should be the TOP priorities of ALL levels of government |
| personal income shouldn't be a factor |
| Try alternative natural remedies instead of manufactured drugs that only benefit the large drug companies. Why not try some of the plants and berries found in Newfoundland that our ancestors have used for years. If they could heal cuts and settle upset stomachs with things found in our forest than maybe it could be used in some other way to treat diseases. |
| Stop any misuse of medical funding in the medical system. |
| Continued research and funding assistance for those with dementia. |
| Our medical system shouldn't be fueled by capitalism. |
| Make sure it is fair.... |
| make them available to most people |
| it may seem senseless to spend big dollars on just one or rare cases but when it is a loved one it is priceless and all lives are valuable. I do not mind paying for more if it would help someone else. But lest be proactive and spend money preventing disease in the first place and make active living affordable and many of teh disease will fade away. |
| Would you fund the drug if it was your Mother or Child? |
| Generic drug options should be available and a system such as India's should be studied and implemented so that our health care dollars are not wasted on large pharmaceutical corporate profits. |
| don't wish to see unnecessary funding of companies that purchase drug companies producing rare drugs then jacking up the price 1000% or more to "recoup their investment". I want to see everyone get the drugs they need, including the option of the "real" drug over the generic, but not at the expense of the entire program |
| drug companies need to be more thoroughly scrutinized about where they get there costs .I think they are highly inflationary ! |
| In taking a survey like this, it is hard not to be selfish with the answers. Perhaps the survey should start with some basic medical condition questions so you see 'who thinks what'... |
| it is sad when a person can't afford the drugs they need to live. this affects everyone. |
| people that really them should get them |
| Reduce the time before generic drugs replace the expensive ones. |
| Ensuring that those who are in low income area have the same care and access to the treatments and drugs as those who are in the high-income. We tend to forget those who are unable to afford treatment for rare diseases. |
| sometimes it's not the funding but are the drugs even available |
| make funding easier to get than so much official papaers thennnn the patient dies waiting in between |
| legal experimental drugs should be covered if it is a last resort and the patient has been educated on the product |
| I feel if it is an expensive drug even when not covered by a health plan the patient should pay some but get help depending on their financial need. |
| This is not an easy thing to comment on as there is only so many resources, but clearly something has to be done. |
| these drugs are only expensive because of the amount of money we pay the employees, If the research or drug company was in a less fortunate place these scientist would be happy that they have a job and that they can happily provide to society no matter what drug for a rare disease they are working on |
| Patients need to be treated as an individual, and decisions should be based on their precise situation. If an expensive drug can help a patient improve their quality of life, even for a short time, it should be considered as a treatment option regardless of cost. |
| Each patient should be treated no matter the cost to the gov't. The gov't has squandered money on many areas other than healthcare, and it's time they made health funding a priority in this country! Health care should be the gov't first concern. |
| Every Canadian should have access to the medications and treatments they need to live a pain free life. |
| I believe rare diseases should receive some kind of break on the cost of drugs even if minimal |
| Do your questions incorporate how rare the disease is? If we can help 0.00001 % of the population for $1M or 0.003% for $100M, we should make democratic decisions to some degree, getting the most bang for our taxpaying dollars. |
| Make all essential drugs free to seniors and those in need |
| Healthcare has exceeded 50% of all taxes. This bloated cost has NOT improved access or reduced wait times.We should set a cap to health care spending to 33% of taxes and allow for the money saved by individual to fund private programs that will reduce demand on the public system. This will introduce a pay for performance model into the system that will drive innovation and reduce waist. |
| Consideration to dispense generic drugs should be undertaken when possible |
| It seems to me that expensive drugs are the result of high R & D costs combined with limited sales opportunities. Does the government provide sufficient tax breaks for the development of these types of drugs? Are the opportunities to purchase insurance which will cover the cost of the drugs? |
| Reduce the time required to approve new drugs |
| How are the drugs funded? Since so much money is raised by non profit organizations for finding cures or developing new drugs, the ROI is already covered by the general public so the medications should be priced accordingly. |
| Do not penalize people that have rare illnesses |
| Fund at least part of the cost would be an option |
| Would like to see as Canadians we help as many people as we can. |
| Put a cap 0n % of profit a company can make on medications!!!!!! |
| This is a tough subject as you have to factor in both compassion and fiscal realities. Often "wonder cures" aren't what they are cracked up to be but how do you put a price on hope? Heartbreaking. |
| please do not tax low income earners |
| Universal health care should be just that.The same for everyone, including access to services medical professionals and reasonably priced prescription drugs. |
| I think that sometimes we have to let some people go/die earlier than they like. We cannot waste money saving everyone especially if they are going to die anyway. Unfortunately sometimes families have to accept that their loved one was unfortunate to get a disease and accept that the many cannot sacrifice for the one. We need to put our resources towards as many people as possible not the sickly few. |
| One should consider the value of a human life in terms of the societal economic and social-technical .contribution an individual makes and should also factor in the age of the individual |
| We should have the option of privately funded health services. |
| i believe Canadians are in denial about the realities of our massive geography!! |
| Require research companies to publicize their costs. |
| Humanity should not be subject to the profiteering of others |
| In order to maintain a quality of health expensive drugs are required and not all are available at reduced costs by provincial health. I pay over 250.00 per month for drugs to keep me breathing. It drains any pension a person may have. |
| I find the so-called 'generic' versions of drugs are not the same as the originals and do not work the same way. Drug plans often do not cover anything other than the 'generics'. This often leaves the public at-risk and feeling like 'second-class citizens' as many are unable to access the drugs they need. |
| I have paid for 12 years into NS's pharmacare, now because of expensive drugs I am facing a huge increase, it hasn't had to pay anything for me, does not seem fair! |
| not to just to lighten load but to cure illness.A lot of money is gathered for cancer but not really for curing it or to make it easier always just for research. |
| availability of generic drugs |
| Is it going to improve the quality of life |
| Important to help the less wealthy people pay for drugs |
| when drugs are only in the US or other parts of the world the cost of these is so expensive that most Canadians can not afford them |
| Reduce the pay of politicians and use the money for funding drugs |
| Make the testing the same in Canada as it is in the US. ie. Lyme Disease. Make Doctors have to be honest about whether something like lyme exists instead of them insisting it doesn't and that the patient is rediculous. Doctors who don't want to help their patient should NOT be doctors. I am appalled that someone I know has a doctor who shrugs his shoulders when asked medical questions. Seriously???? |
| without healthcare nothing else matters |
| consideration must be given to examine why drugs are so expensive and ways to decrease the costs |
| consider the income level of each patient; determine insurance available both in-Canada and out-of Canada sources for each patient - some patients could have health insurance paid from employment in foreign countries. Might look at negotiating payments with the other Country reimbursement programs, such as retiree health insurance not payable out of the patient's country of retirement. |
| All Seniors in Canada should NOT have to pay a deductible like I do in MB, based on a % of my income. As a Type 2 Diabetic my costs run more than $2000 a year, before I receive any drugs free. I have to make a decision, some months-do I purchase way overprice B/G Test Stripes ($90. a box of 100)or do I use my meager income for food. It is a shame that Seniors in Canada are treated as 2nd class citizen by all levels of Government. |
| drugs are a band-aid.. closed hospitals and a shortage of nurses and doctors are a real problem here. so tired of seeing signs that say our dr.s are not taking new patients. what good is a new drug if there's no dr. to dispense it. |
| Whether or not the drug is going to extend life at least 5 years. |
| National government control should provide cost savings. |
| Are there organic options available to some treatments? |
| Pharmaceuticals are the least expensive means of treating a vast variety of health care problems and should not be relegated to the back shelf simply because of cost ! |
| Actual cost of the drug versus what is being charged. |
| Health Care in Canada must be fair, it must be available to all Canadians. Treatments for those with a rare disease must be paid for by the Government to prolong life.Thank you for your time. |
| Somehow the physicians need to be held accountable for their treatment plan(s), Giving drugs just because they can does not serve anyone. Likewise, if there is a rare condition and there is a proven treatment that dramatically improves the quality of life, then that should be given. I'm convinced there is room in the health care system for these approaches because there is so much waste with different Doctors repeating the same procedures on the same person, the costs associated with poor diet and lack of exercise prevent allowing those in real need the medical help they need, and just better use of funds would realize savings that could be redirected to rare diseases. With many more mixed-multicultural marriages I'm sure that rare conditions will appear and there will need to be a way to address how to care for the diseases that result. |
| Will the expensive drugs really help in reducing pain and help heal ??? |
| Quality health care is not just for the rich but should be equally available for all. |
| Severity of the patients disease and impact on quality of life should be a priority |
| I understand that developing new drugs can be expensive but I don't believe drug companies should be allowed to make gross profits at the expense of suffering people. |
| Research into alternatives that are less expensive and equally or more effective |
| There should be consideration for the potential of contributing to society by the individual (expensive drug to combat/treat a disease which primarily affect youth should be given higher consideration than drugs to treat diseases of the aged. |
| Since rare diseases and such are "rare" by definition then people should have equal access across Canada to the drugs that help in these situations. More helpful drugs would be used and more people would be helped. Doing this may make everything more affordable for everyone as well. The drug makers could lower their prices because they are selling more and patients wouldn't have to pay as much individually. |
| The impact it will have on the most people |
| Because of the advancements in health care, it is time to set priorities.I do not believe that ALL interventions are positive. Babies less than one pound (500 gms) should not be saved as they create incredible costs during their lifetime. Pregnant women should NOT have access to 3 or 4 ultrasounds during a pregnancy, Not all new technology has a positive effect on personal health. |
| We must find a way to wrestle the administrative and bureaucratic costs out of health treatment!!! |
| I DO NOT BELIEVE WE CAN COVER COSTS OF ALL NEW DRUGS. |
| expensive rare disease costs for individuals has to be weighed against the availability of funds to service care for everyone |
| it would be appropriate to consider whether the patient is responsible for his/her need for expensive drugs |
| Fund it if it is effective in treating patients |
| I think this is an important initiative - otherwise people with these diseases and their families continue to be marginalized |
| Equally available for all not just what the "rich" can afford |
| The force of the federal Government should be used to prevent profiteering by drug companies |
| If a patient needs a specific drug they should have it regardless of cost |
| I think if there was a drug available to cure an disease, then we should have it avail to us, in Canada, if it meant saving lives then if its too expensive for Govt then it should be at the least be paid for half of the cost |
| need legislation to allow drug companies to research treatments for rare diseases but with limits on related profits |
| It is most important to remember those with rare illnesses/conditions... |
| The age of the patient as well as the prognosis. |
| Expensive drugs take away from the majority to benefit a few. That is not fair to the majority. Policy makers must make decisions for the majority and contains costs. |
| Just try and help whoever needs it. |
| My belief is that drug companies are taking advantage of the public funding of our health system and charging us excessively.  The government needs to seriously challenge the charges. |
| all necessary drugs should be covered |
| Force the patient and private insurance to share some of the cost. |
| Children should not be given priority over adults in my opinion. |
| Only that drugs should be judged by effectiveness |
| If you can somehow get government on-board to stop pharmaceutical companies from over-charging and provide life-saving medication to people who need it. We are being gouged and the government is allowing it to happen. That is why you are doing this survey. There needs to be some regulation...but there never will be...as long as the government has its hand in the coffer. |
| Why did the health department of Ontario increase Zoladex Depot LA 10.8 mg by 129.$ for cancer Patients |
| Funding expensive drugs should also be weighed against to what extent they will improve the quality of life. |
| Prevention and the ability for exercise/food choices to improve conditions where possible, rather than expensive drugs. |
| free drugs for everyone. earning less than 50k a year |
| Government should be smart when spending public money on health issues! |
| Perhaps the drug companies can be a little more compassionate and lower costs to make them available to more people |
| When you are sick you can't work so to have a drug expense is the last worry you need. |
| A lot of people can't afford medications and like me, they don't have any insurance because we can't afford it. It would be nice if the Senior's program would cover more drugs without us having to wait for the special authorization to go through. My husband is a very sick man and sometimes we have to wait a week or two to get a new medication, just not fair. |
| Why not have drugs bought in bulk and distributed to the provinces.  It is most important that health alternatives to drugs be approved and funded. Drug companies don't want people to heal; they want them to continue taking drugs. |
| what about alternate ways to do health care? chiropactic, physio, emotional, vitamins, etc....... |
| strong advocacy to keep drug companies from gouging the vunerable. From what I hear is that they often raise the cost way beyond what the research and cost of the ingredient is. And there should be one supplier so that large purchases can reduce costs. |
| Yes. What about the cost to the Health care system if you don't fund them ? |
| Patients should be given more freedom to shape their health plans. |
| RIght now drugs of any kind for seniors are limited to their financial ability to pay for them....the most vulnerable in our society with (in most cases) the lowest income of their lives, do not quality for assistance for any type of drug, whether generic or rare and expensive |
| I think that some of them should be based on income levels and whether or not the person has a health plan. |
| If an alternative drug/treatment is not available then the drug should be provided if it means saving the person's life - an investigation should be launched into the profits of drug companies and their pretense of not being able to provide these drugs at lower cost to either individuals or the health care system in general. |
| It is the fact that we are all Canadians & I believe that Health Care should be the same across this Nation & governed from Ottawa |
| people should not have to pay more for the treatment required |
| Consider any alternatives and if none then we have to look after these people and do it. |
| Will the costs come with guarantees of a cure or comfort for patients with rare diseases? |
| Make sure that the people who need the drugst get it quickly! |
| I think Patients should bear some of the cost of expensive drugs.. within their means.. the government can't continue to pay for everything for everybody |
| FUNDING IS IMPORTANT BUT THE NEED FOR TREATMENT IS PARAMOUNT |
| * an important consideration is the ability for individuals to afford payment for the drugs. As an affluent individual, I would not expect that I would be heavily funded for drugs versus someone who might be classed as low income or living at or below the poverty line. However, that is not to say that I should not receive any funding but that the level of funding should reflect my ability to pay, providing that it does not decimate my savings or ability to support myself and my family. |
| It's not just "expensive drugs" that lack funding in this country. There are a whole spectrum of diseases which are not recognized by Health Canada. And because they have no recognition, the drugs to treat them - which are usually readily available - are not covered by any health plan. |
| It is the price of these expensive drugs compared to other countries that concerns me. How is it possible for me to pay $100 for a drug in Canada and only $10 for the exact drug in India? |
| please consider insurance to cover the cost |
| My health care insurance pays for expensive drugs so I'm biased but I do pay a premium for this service. |
| should be more affordable for seniors |
| Without the development of expensive drugs how do you move forward and improve drug treatment so that more generic drugs can be also developed. As well, drugs developed for one type of disease can often be used in treatment of other diseases (at least that is what I understand) |
| A persons life has to be more important than their ability to pay |
| I believe that each person should have the same drugs / treatment available to them. It shouldn't matter what province you are from or what your income is. If you are a Canadian citizen then whats available to one should be available to all. When a person is down (sickness) I think they deserve all the help they can get from the government. |
| The drug companies are screwing the negotiating process in Canada and our negotiators aught to ensure a more worldwide purchasing policy with equal pricing of all life saving drugs. |
| Il faut avoir des bonnes probabilités de guérison et/ou de prolongement avec qualité de la durée de vie du patient. |
| si la vie du patient en dépend, il faut lui payer |
| rapidité ds soins |
| eduire le salaire des burocrate afin davoir plus de financement pour les medicaments cher pour ceux qui en ont vraiment besoin |
| les assurances de la personne |
| il faut voir si le patient prefere etre soigne ou beneficier de l aide medicale a mourir |
| tous les medicaments ne peuvent être disponible à tous. Je suggère que les gouvernements l'expliquent comme une asssurance publique, déductible si excès d'utilisation, maximum de coût par patient, de traitements, bref gérer nos $ en bon père de famille. |
| Peu importe la maladie, le traitement doit être analysé équitablement pour tous. On débute par celui le moins cher et on change vers le plus cher au besoin. Personne ne devrait avoir à choisir entre sa qualité de vie, la mort ou payer ses médicaments. Dans notre société, nous devrions être capable d'absorber ces coûts collectivement. |
| Mettre à profit sous forme d'imposition un fond indépendant par les mieux nanti de la société grande société ou autres qui n'affecteraient en rien leurs qualité de vie !! |
| Les revenus de la personne/famille. |
| Le rapport coûts/bénéfices |
| le revenu personnel |
| améliorer la qualité de vie des patient. |
| Maximiser la convivialiter entre chaque intervenant du système de santé afin que le patient se retrouve au centre du sujet,non pas a la fin du sujet comme présentement.je trouve la priorité (patient)très loin des objectifs fixer par nos pratiquants et nos élus. |
| Je crois qu'il ne devrait pas y avoir de discrimination quant au financement des méd. chers. La souffrance et la qualité de vie n'ont pas de prix pour une société. |
| leur pourcentage de réussite |
| Selon moi la santé est de domaine provincial, inclure le fédéral est une grave erreur |
| Créer un programme national d'achat en bloc des médicaments et rendre tout abus par des exploiteurs de la misère humaine illégal. |
| Diminuer la bureaucratie. C'est là que ça coûte cher. il y a trop d'administrateurs pour le nombre d'exécutant (ceux qui donnent les soins) |
| Tout dépend de l'efficacité du médicament, je crois. |
| L'âge moyen des personnes atteintes |
| Je suis d'accord pour aider les personnes qui ont des maladies rares et qui ont besoin de médicaments chers pour lutter contre leur maladie. |
| le prix desmédicaments génétique |
| Accentuer le message sur la prevention et inciter les personnes a avoir un comportement et une mode de vie sains |
| Valorisé les médicaments générique au lieu d'enrichir les grosses compagnies. |
| les mettre moins dispendieux pour les ainés |
| Un gouvernement a toujours le loisir (même si ce n'est pas éthique) de commander quelques copies d'originaux pour vérifier la probabilité d'en faire des génériques. |
| Tous les gouvernements doivent prendre les mesures pour le contrôle de tous les médicaments sans exception quitte à choisir de financer les médicaments les moins chers toutes autres choses étant égales par ailleurs. |
| efficacité du produit hors de tout doute |
| penser a l'acces au soin pour tous et a l'acces aux medicaments en diminuant les prix |
| S'assurer que le médicament nouveau et cher a des effets significativement plus efficaces que tout médicament actuellement dispnible |
| L'âge et l'état de santé global de la personne |
| Chance de succès de guérison Volonté et décision du patient |
| Dans un contexte d'austérité, bien sur, il faut regarder toutes les facettes du problème sans oublier que le patient demeure le principal sujet dans un tel dossier. |
| Pourquoi mettre en compétition les médicaments chers lorsqu'on en a besoin avec les autres. Tous doivent avoir accès aux médicaments dont ils ont besoin quelque soit le prix. |
| Prioriser la santé dans le budget |
